# *BRAF*^*V600E*^ mutation is highly prevalent in thyroid carcinomas in the young population in Fukushima: a different oncogenic profile from Chernobyl

**DOI:** 10.1038/srep16976

**Published:** 2015-11-20

**Authors:** Norisato Mitsutake, Toshihiko Fukushima, Michiko Matsuse, Tatiana Rogounovitch, Vladimir Saenko, Shinya Uchino, Masahiro Ito, Keiji Suzuki, Shinichi Suzuki, Shunichi Yamashita

**Affiliations:** 1 Department of Radiation Medical Sciences, Atomic Bomb Disease Institute, Nagasaki University; 2 Department of Health Risk Control, Atomic Bomb Disease Institute, Nagasaki University; 3 Department of Global Health, Medicine and Welfare, Atomic Bomb Disease Institute, Nagasaki University; 4 Nagasaki University Research Centre for Genomic Instability and Carcinogenesis (NRGIC); 5 Department of Thyroid and Endocrinology, Fukushima Medical University; 6 Department of Pathology, Nagasaki Medical Centre; 7 Department of Surgery, Noguchi Hospital

## Abstract

After the accident at the Fukushima Daiichi Nuclear Power Plant, the thyroid ultrasound screening program for children aged 0–18 at the time of the accident was started from October 2011. The prevalence of thyroid carcinomas in that population has appeared to be very high (84 cases per 296,253). To clarify the pathogenesis, we investigated the presence of driver mutations in these tumours. 61 classic papillary thyroid carcinomas (PTCs), two follicular variant PTCs, four cribriform-morular variant PTCs and one poorly-differentiated thyroid carcinoma were analysed. We detected *BRAF*^*V600E*^ in 43 cases (63.2%), *RET/PTC1* in six (8.8%), *RET/PTC3* in one (1.5%) and *ETV6/NTRK3* in four (5.9%). Among classic and follicular variant PTCs, *BRAF*^*V600E*^ was significantly associated with the smaller size. The genetic pattern was completely different from post-Chernobyl PTCs, suggesting non-radiogenic etiology of these cancers. This is the first study demonstrating the oncogene profile in the thyroid cancers discovered by large mass screening, which probably reflects genetic status of all sporadic and latent tumours in the young Japanese population. It is assumed that BRAF^V600E^ may not confer growth advantage on paediatric PTCs, and many of these cases grow slowly, suggesting that additional factors may be important for tumour progression in paediatric PTCs.

After the accident at the Fukushima Daiichi Nuclear Power Plant due to the Great East Japan Earthquake occurred on March 11^th^, 2011, there have been great concerns regarding a possible health impact by radioactive materials released into the environment. Fukushima prefecture, therefore, started the Fukushima Health Management Survey for long-term health management^1^,^2^. This survey includes the thyroid ultrasound screening program for all children aged 0–18 years old at the time of the accident. After the Chernobyl accident, internal exposure to radioactive iodine induced a massive increase in childhood thyroid cancers in Belarus, Ukraine and Russia^3^,^4^,^5^,^6^. This increase started to appear 4–5 years after the accident. Therefore, the first round of the screening program, so-called the baseline survey, was started in October, 2011 to grasp the general ultrasound findings in children and adolescents, because such a screening program for a huge number of young individuals using advanced ultrasound technologies has never been done. After the first round of the screening, the prevalence of thyroid cancers in Fukushima appeared to be quite high: 84 per 296,253 children as of October 2014 (http://www.fmu.ac.jp/radiationhealth/results/media/17-2_Thyroid_Ultrasound_Examination_I-S.pdf). Most of them were papillary thyroid carcinomas (PTCs). Logically, this increase has been thought to be due to screening effect with advances in diagnostic instruments in recent years, because the estimated thyroid doses of radioiodine exposure in Fukushima were far lower than those in Chernobyl^7^,^8^,^9^, and the period from the date of the accident until the onset is too short. Presumably, a real morbidity rate may be comparable throughout Japan^10^,^11^, and many of the PTC cases could be silent or very slowly progressing until middle or older age.

PTCs have particular genetic alterations such as *RET/PTC* rearrangements and point mutations in the *BRAF* and *RAS* family genes resulting in activation of the mitogen-activated protein kinase (MAPK) pathway^12^,^13^. There are more than 15 different types of *RET/PTC* chimeric genes that differ according to the 5’ partner gene involved in the rearrangement. *RET/PTC1* and *RET/PTC3* are the most common types, accounting for more than 90% of all the rearrangements. These oncogenes rarely overlap in a same tumour, providing strong genetic evidence for the requirement of constitutive active MAPK signaling for the development of PTC. In adult sporadic PTCs, the *BRAF*^*V600E*^ mutation is the most prevalent genetic alteration ranging approximately 30–70%^14^,^15^,^16^,^17^,^18^. On the other hand, the *RET/PTC* rearrangements have been reported to be dominant in childhood sporadic PTCs^19^,^20^. Regarding radiation-induced thyroid carcinomas, post-Chernobyl PTCs also had higher prevalence of the *RET/PTC* rearrangements. Especially, in cases with short latency (developed less than 7–10 years after the accident), they were found in 64–86% of the tumours, and *RET/PTC3* was the most frequent type of the rearrangement in this group^5^. Consequently, the pattern of genetic alterations in PTCs depends on patient age and etiology of the tumours.

In the present study, we investigated the presence of various known genetic alterations in 68 operated cases discovered in Fukushima. First, we aimed to assess whether their genetic pattern is similar to that of post-Chernobyl PTCs, even though these Fukushima cases are very unlikely to be radiation-induced. Second, we also attempted to gain insight into the carcinogenic mechanisms of the thyroid cancers found by the large mass screening program in the young population in Fukushima.

## Results

### The profile of the genetic alterations

We obtained tissue samples from 68 patients operated at Fukushima Medical University, consisting of 61 classical PTCs, two follicular variant PTCs, four cribriform-morular variant PTCs and one poorly differentiated thyroid carcinoma (PDTC). We first screened them for *BRAF* (exon 15), *HRAS*, *KRAS* and *NRAS* (codons 12, 13 and 61), and *RET/PTC1* and *RET/PTC3*. In samples without above-mentioned frequent known genetic alterations, we next investigated the presence of *AKAP9/BRAF* and *ETV6(exons 4 and 5)/NTRK3* rearrangements. The mutation in the *TERT* promoter (C250T and C228T) was checked in all samples. The clinicopathological features and genotyping results are summarized in Table 1 and 2, respectively. We found these known oncogenes in 54 out of 68 (79.4%) samples. The *BRAF*^*V600E*^ mutation was highly prevalent and found in 43 out of 68 (63.2%). Note that four cases showed the weak signal of a mutant allele: the intensity of A signal in the sequence chromatogram was less than 20% compared to that of T signal (Table 1). In these tumours, the *BRAF*^*V600E*^ mutation may not be clonal. None of samples had the *RAS* mutation nor the *TERT* promoter mutation. Regarding the fusion genes, six *RET/PTC1*, one *RET/PTC3* and four *ETV6(ex.4)/NTRK3* were detected. *AKAP9/BRAF* and *ETV6(ex.5)/NTRK3* were not found. In one PDTC, we did not find any of the above genetic alterations. The four cases with cribriform-morular variant PTCs are highly suspected to be familial adenomatous polyposis; the analyses of the *APC* gene/genetic counseling are being conducted, and their results will be published elsewhere.

### Clinicopathological correlations

We compared clinicopathological parameters between the *BRAF*^*V600E*^ mutation-positive PTC cases and the *BRAF*^*V600E*^-negative PTCs including the fusion gene-positive and mutation-negative cases, excluding cribriform-morular PTCs. As shown in Table 3, the *BRAF*^*V600E*^ mutation was significantly associated with smaller tumour size (12.2 ± 6.8 mm in the *BRAF*^*V600E*^-positive PTCs vs. 18.3 ± 9.5 mm in the *BRAF*^*V600E*^-negative PTCs) but not with the TNM parameters and extrathyroidal extension in univariate analysis. On multivariate analysis adjusted for age and sex, tumour size in *BRAF*^*V600E*^-positive cases was still significantly smaller than that in the negative cases (Table 3), and also an association with older patients’ age was detected after adjustment for sex (Table 3).

## Discussion

In the present study, we have demonstrated that high prevalence of the *BRAF*^*V600E*^ mutation and low frequency of chromosomal rearrangements such as *RET/PTC* in young PTC cases were found by the screening program. There are not so many publications about the *BRAF* mutation in paediatric thyroid carcinomas. In the early period (2004–2005) after the discovery of the *BRAF* mutation in thyroid cancers, its prevalence in paediatric PTCs was reported to be 0–20%^21^,^22^,^23^,^24^. However, two very recent papers published in 2014 demonstrated much higher prevalence (63 and 36.8%, respectively), especially in tumours with classic papillary subtype (71.4 and 63.6%, respectively)^25^,^26^. We cannot explain this discrepancy although all of these studies analysed a limited number of cases. One possibility is that this increase may be due to improved sensitivity of detection methods: PCR-restriction fragment length polymorphism (RFLP) and pyrosequencing were used in the recent studies. As the histological classification of most of our Fukushima cases was classic papillary subtype, our data are consistent with the recent studies. The prevalence of the *BRAF*^*V600E*^ mutation depends on population and iodine intake^27^. In East Asian countries such as South Korea and Japan where iodine intake is very high, PTCs usually show very high rate of the *BRAF*^*V600E*^ mutation. Our cases in Fukushima demonstrated a similar rate. In terms of the *BRAF* mutation in radiation-induced paediatric thyroid carcinomas including post-Chernobyl PTCs, its prevalence was reported to be low (0–17%)^5^,^21^,^28^. Furthermore, point mutations have been shown to display negative dose-response relationship in thyroid cancers in Chernobyl^29^ and in A-bomb survivors^30^. These observations are suggestive of non-radiogenic etiology of the thyroid cancers in Fukushima.

In the post-Chernobyl paediatric PTCs, the *RET/PTC* rearrangements were usually detected in more than 50%. *RET/PTC3* was the most common type in tumours with short latency (4–8 years after the exposure), whereas tumours developed after longer period of time had *RET/PTC1* predominantly. In addition, *RET/PTC3* had a strong correlation with solid variant subtype, while *RET/PTC1* usually found in classic papillary subtype^5^. Ricarte-Filho *et al.*^31^ reported the results of analysis of driver mutations in post-Chernobyl radiation-exposed PTCs that showed a similar age range to our series (radiation-exposed, median: 17.8 years old, range: 13.4–23.0; Fukushima, median: 18.0 years old, range: 9–22). In this report, the prevalence of the *RET/PTC* rearrangements in the radiation-exposed cases was 57.7%. The detected rate of the *RET/PTC* rearrangements in our series was only 10.3%, all of which were *RET/PTC1* except one *RET/PTC3*, also suggesting that the mode of carcinogenesis in the Fukushima cases is different from post-Chernobyl PTCs.

The prevalence of the *RET/PTC* rearrangements in sporadic paediatric PTCs was reported to be also high (30–67%)^5^,^19^. In the above paper by Ricarte-Filho *et al.*^31^, sporadic PTCs, most of which were teenagers (median: 16.6 years old, range 5.8–19.8), were also analysed, and the prevalence of the *RET/PTC* rearrangements was 25.9%. As far as we are aware, there are only two studies that analysed Japanese cases, and they demonstrated 30% (9–14 years old) and 41.9% (<20 years old)^32^,^33^. However, note that the cohort in the present study is completely different from those in the above previous studies because the data in this work were obtained due to mass screening of basically healthy individuals without any symptom. Even though the incidence in Fukushima has been much higher than that reported previously, it is quite unlikely that this is due to radiation exposure because of their shorter latency and higher age distribution^34^,^35^. Moreover, the patterns of the genetic alterations in our cases were completely different from radiation-induced PTCs. Assuming that most of these cases would be silent until middle or older age, we could speculate that paediatric PTCs with the *BRAF*^*V600E*^ mutation may be not so progressive compared to those with the rearrangements. Consistent with this hypothesis, our data in the present study has demonstrated that tumours with the *BRAF*^*V600E*^ mutation were significantly smaller than *BRAF*^*V600E*^-negative cases including *RET/PTC* or *ETV6/NTRK3*. It is supposed that we detected these tumours earlier in life because of the extremely sensitive ultrasound screening procedure.

*ETV6/NTRK3* has been recently found in both radiation-associated paediatric PTCs and sporadic paediatric PTCs with comparable frequencies (2 of 26 in exposed and 2 of 27 in sporadic)^31^, although the number of analysed cases was limited. It has been reported that this rearrangement was significantly more common in post-Chernobyl PTCs (mean age: 22.7 ± 5.1 years; 9 of 62, 14.5%) than in sporadic PTCs (mean age: 45.6 ± 17.7 years; 3 of 151, 2%); however, the age was not matched in this study^29^. Thus, the association between *ETV6/NTRK3* and radiation exposure is not clear, and this fusion gene may just be associated with young age.

*TERT* promoter mutations (C250T and C228T) have also been discovered recently in thyroid cancers^36^,^37^. These mutations are highly associated with older age and worse prognosis^38^,^39^. Some papers have also reported that these mutations are correlated with the presence of the *BRAF*^*V600E*^ mutation^36^,^38^,^39^,^40^. Here we demonstrate that these mutations were completely absent in this age group even though many of them carried the *BRAF*^*V600E*^ mutation.

In conclusion, the genetic profile of the Fukushima thyroid cancers was completely different from post-Chernobyl radiation-induced PTCs, suggesting non-radiogenic etiology of these cancers. However, note that since similar high-resolution ultrasound apparatus was not available after the Chernobyl accident, it might be difficult to strictly compare the results of the two populations. Simultaneously, we for the first time demonstrate the distribution of the oncogenes in the young thyroid cancer cases discovered by large mass screening. This probably reflects genetic status of all sporadic and latent thyroid carcinomas in the young Japanese population. The prevalence of the *BRAF*^*V600E*^ mutation was comparable to Japanese adult cases, implying that the carcinogenic mechanisms may be similar between paediatric and adult PTCs. However, it is assumed that the *BRAF*^*V600E*^ mutation may not confer growth advantage on paediatric PTCs, and many of these tumours do not grow rapidly, suggesting that additional factors may be necessary to enhance tumour growth. Thus, it is beneficial to identify such a key factor to improve the management of paediatric PTC patients.

## Methods

The thyroid ultrasound screening program under the Fukushima Health Management Survey is being conducted by Fukushima Medical University. This program covers all residents in the Fukushima prefecture aged from zero to 18 years old at the time of the accident. During first three years, the baseline survey has been conducted. We also included three cases in the same age range incidentally discovered through other medical examinations in the same region.

We analysed 68 thyroid cancer cases operated between February 2013 and September 2014 (mean age: 17.3 years old; mean tumour size: 14.7 mm; 22 males and 46 females) at Fukushima Medical University. Fresh tumour tissue samples were obtained during surgical operations, snap-frozen in liquid nitrogen, and stored at −80 °C. DNA and total RNA were extracted at the same time using ISOGEN reagent (Nippon Gene) according to the manufacturer’s protocol. Mutations of *BRAF* (exon 15), *HRAS*, *KRAS* and *NRAS* (codons 12, 13 and 61), and *TERT* promoter (C250T and C228T) were examined by direct DNA sequencing. PCR enzymes and primer sequences used for PCR amplifications and sequencing are listed in Supplemental Table S1. PCR products were then treated with ExoSAP-IT PCR clean-up reagent (GE Healthcare), and sequencing was performed with Big Dye Terminator sequencing kit version 3.1 (Applied Biosystems) on an ABI3730 automated sequencer (Applied Biosystems). For *RET/PTC*, *AKAP9/BRAF* and *ETV6/NTRK3* detection, total RNA was reverse transcribed using High Capacity RNA-to-cDNA kit (Applied Biosystems). Subsequent PCR amplifications were done using PCR enzymes and primers listed in Supplemental Table S1. We included 3–5 negative controls (w/o template) per 96 samples in every PCR to ensure contamination-free amplifications.

Statistical analysis was performed using the SPSS software version 21.0.0.0 (IBM). Mann-Whitney or Fisher’s exact test were used for continuous variables or group analyses, respectively. For multivariate analyses, linear or logistic regression models were used. The *p* value less than 0.05 was regarded as indicating statistical significance.

This study was approved by the ethics committees of Fukushima Medical University and Nagasaki University. Written informed consent was obtained from each patient. All experiments were carried out in accordance with the approved study plan and relevant guidelines.

## Additional Information

**How to cite this article**: Mitsutake, N. *et al.* *BRAF*^*V600E*^ mutation is highly prevalent in thyroid carcinomas in the young population in Fukushima: a different oncogenic profile from Chernobyl. *Sci. Rep.* **5**, 16976; doi: 10.1038/srep16976 (2015).

## Supplementary Material

Supplementary Information

## Figures and Tables

**Table 1:** Clinicopathological features of the current cases.

Age at operation (ave, y.o. ± s.d.)	17.3 ± 2.8
(median: range, y.o.)	18: 9–22
Sex	
M	22
F	46
Tumor size (mm ± s.d.)	14.7 ± 9.2
Histology	
CP	61
FV	2
CMV	4
PD	1
pT	
pT1 or 2	37
pT3	31
pN	
pN0	15
pN1a or 1b	52
M	
M0	65
M1	2
pEx	
pEx0	36
pEx1	32

CP: classic papillary, FV: follicular variant, CMV: cribriform-morular variant, PDTC: poorly differentiated.

**Table 2:** Genetic alterations in the current cases.

*BRAF*^*V600E*^	43 (63.2%)*
*HRAS*	0
*KRAS*	0
*NRAS*	0
*RET/PTC1*	6 (8.8%)
*RET/PTC3*	1 (1.5%)
*ETV6(ex4)/NTRK3*	4 (5.9%)
*ETV6(ex5)/NTRK3*	0
*AKAP9/BRAF*	0
*TERT C250T*	0
*TERT C228T*	0

^*^including 4 cases in which the strength of A signal in the sequence chromatogram was less than 20% compared to that of T signal.

**Table 3:** Association of the *BRAF* ^*V600E*^ mutation with clinicopathological features of the PTC cases found by mass screening.

	*BRAF*^*V600E*^	Rearrangement or unknown mutation	Univariate p-value; OR (95% CI)	Multivariate p-value; OR or B (95% CI)
All PTC cases	43	20		
Age at operation (y.o. ± s.d.)	18.2 ± 2.3	16.7 ± 3.7	0.115^a^	**0.041; B** **=** **1.629 (0.068–3.190)**^c^
Sex			0.155^b^; OR = 2.880 (0.823–10.076)	0.089; OR = 3.115 (0.841–11.494)^e^
M	18	4		
F	25	16		
Tumor size (mm ± s.d.)	12.2 ± 6.8	18.3 ± 9.5	**0.002**^a^	**0.005; B** **=** **-6.547 (−11.060–−2.034)**^d^
Microcarcinoma			**0.012**^b^**; OR** **=** **5.937(1.515–23.260)**	**0.037; OR** **=** **4.477 (1.095–18.303)**^f^
≤10 mm	22	3		
>10 mm	21	17		
Histology			0.097^b^; OR = 11.757(0.537–257.210)	Not performed because of no FV in the *BRAF*^*V600E*^ group
CP	43	18		
FV	0	2		
pT			0.103^b^; OR = 2.840(0.942–8.566)	0.086; OR = 2.779 (0.866–8.923)^f^
pT1 or 2	26	7		
pT3	17	13		
pN			0.714^b^; OR = 0.667(0.165–2.692)	0.783; OR = 0.800 (0.164–3.917)^f^
pN0	6	4		
pN1a or 1b	36	16		
M			0.090^b^; OR = 0.080(0.004–1.764)	Not performed because of no M in the *BRAF*^*V600E*^ group
M0	43	17		
M1	0	2		
pEx			0.109^b^; OR = 0.388(0.129–1.166)	0.096; OR = 0.370 (0.115–1.193)^f^
pEx0	18	13		
pEx1	25	7		

^a^Mann-Whitney test.

^b^Fisher’s exact test.

^c^Linear regression adjusted for sex.

^d^Linear regression adjusted for age and sex.

^e^Logistic regression adjusted for age.

^f^Logistic regression adjusted for age and sex. OR: odds ratio, B: regression coefficient, CI: confidential interval, CP: classic papillary, FV: follicular variant.
