# Supplementary material for: BRAFV600E mutation is highly prevalent in thyroid carcinomas in the young population in Fukushima: a different oncogenic profile from Chernobyl
Source: Sci Rep. 2015 Nov 20;5:16976. doi: 10.1038/srep16976 (PMC4653756; doi:10.1038/srep16976)
Supplement: Supplementary Information [file srep16976-s1.pdf]

Supplementary Information

BRAF^{V600E} **mutation is highly prevalent in thyroid carcinomas in the young population in Fukushima: a different oncogenic profile from Chernobyl**

Norisato Mitsutake[1,4]†*, Toshihiko Fukushima[5]†, Michiko Matsuse[1]†, Tatiana Rogounovitch[3], Vladimir Saenko[2], Shinya Uchino[7], Masahiro Ito[6], Keiji Suzuki[1], Shinichi Suzuki[5]§*, Shunichi Yamashita[1,2]§

[1]Department of Radiation Medical Sciences, [2]Department of Health Risk Control, [3]Department of Global Health, Medicine and Welfare, Atomic Bomb Disease Institute, Nagasaki University

[4]Nagasaki University Research Centre for Genomic Instability and Carcinogenesis (NRGIC)

[5]Department of Thyroid and Endocrinology, Fukushima Medical University

[6]Department of Pathology, Nagasaki Medical Centre

[7]Department of Surgery, Noguchi Hospital

†, §These authors contributed equally.

*Correspondence to:

Norisato Mitsutake, MD PhD, E-mail: mitsu@nagasaki-u.ac.jp

Shinichi Suzuki, MD PhD, E-mail: shsuzuki@fmu.ac.jp

This PDF file includes:

Supplementary Table S1. PCR enzymes and primer sequences

Supplemental Table S1. PCR enzymes and primer sequences

Gene	Primer sequence	Annealing Temperature (ºC)	Amplicon size (bp)	PCR enzeme	Reference
BRAF(ex15)	5'-ACATACTTATTGACTCTAAGAGGAAAGATGAA-3'<br>5'-GATTTTTGTGAATACTGGGAACTATGA-3'	60	400	KOD FX	
H-RAS(cdn12)	5'-AGCAGGGCCCTCCTTGGCAG-3'<br>5'-CAGCCAGCCCTATCCTGGCTG-3'	65	261	AmpliTaq	
H-RAS(cdn61)	5'-CAGGGAGAGGCTGGCTGTGTG-3'<br>5'-CCACCTGTGCGGCGTGGGCT-3'	65	298	ExTaq	
K-RAS(cdn12)	5'-GGTACTGGTGGAGTATTTGATAGT-3'<br>5'-CTCATGAAAATGGTCAGAGAAACCT-3'	60	290	ExTaq	
K-RAS(cdn61)	5'-GGTGCACTGTAATAATCCAGACTG-3'<br>5'-CTATAATTACTCCTTAATGTCAGCTT-3'	60	268	ExTaq	
N-RAS(cdn12)	5'-CACACTAGGGTTTTCATTTCCATTG-3'<br>5'-GGTAAAGATGATCCGACAAGTGAG-3'	63	283	ExTaq	
N-RAS(cdn61)	5'-TTGAACTTCCCTCCCTCCCTGC-3'<br>5'-AGCTCTATCTTCCCTAGTGTGGTAA-3'	65	316	AmpliTaq	
RET/PTC1	5'-GCCTGGAGGACCTCACCAA-3'<br>5'-CTCTGCCTTTCAGATGGAA-3'	56	255	AmpliTaq	
RET/PTC3	5'-ACCTGCCAGTGGTTATCAAGC-3'<br>5'-TTCGCCTTCTCCTAGAGTTTTTCC-3'	59	150	AmpliTaq	
TERT prom	5'-CAGCGCTGCCTGAAACTC-3'<br>5'-GTCCTGCCCCTTCACCTT-3'	63	163	KOD FX	Melo *et al.*
AKAP9/BRAF	5'-AGCAAGAACAGTTGATTTTGGA-3'<br>5'-GCAGACAAACCTGTGGTTGA-3'	63	181	KOD FX	Ciampi *et al.*
ETV6(ex4)/NTRK3	5'-ACAGCCGGAGGTCATACTGCAT-3'<br>5'-TTGTGTCCCTGACGGAAGTACTG-3'	68	207	KOD FX	
ETV6(ex5)/NTRK3	5'-AAGCCCATCAACCTCTCTCA-3'<br>5'-TCCTCACCACTGATGACAGC-3'	60	139	AmpliTaq	Leeman-Neil *et al.*

References:
Melo, M. *et al.* TERT promoter mutations are a major indicator of poor outcome in differentiated thyroid carcinomas. *J. Clin. Endocrinol. Metab*. **99**, E754-765 (2014).
Ciampi, R. *et al.* Oncogenic AKAP9-BRAF fusion is a novel mechanism of MAPK pathway activation in thyroid cancer. *J. Clin. Invest.* **115**, 94-101 (2005).
Leeman-Neill, R. J. *et al.* ETV6-NTRK3 is a common chromosomal rearrangement in radiation-associated thyroid cancer. *Cancer* **120**, 799-807 (2014).
